# Financing Universal Coverage in Malaysia: a case study

**DOI:** 10.1186/1471-2458-12-S1-S7

**Published:** 2012-06-22

**Authors:** Hong Teck Chua, Julius Chee Ho Cheah

**Affiliations:** 1 Performance Management and Delivery Unit (PEMANDU), Prime Minister’s Department, Malaysia; 2 Global Public Health, Jeffrey Cheah School of Medicine and Health Sciences, Monash University Sunway Campus, Malaysia

## Abstract

One of the challenges to maintain an agenda for universal coverage and equitable health system is to develop effective structuring and management of health financing. Global experiences with different systems of health financing suggests that a strong public role in health financing is essential for health systems to protect the poor and health systems with the strongest state role are likely the more equitable and achieve better aggregate health outcomes. Using Malaysia as a case study, this paper seeks to evaluate the progress and capacity of a middle income country in terms of health financing for universal coverage, and also to highlight some of the key underlying health systems challenges.

The WHO Health Financing Strategy for the Asia Pacific Region (2010-2015) was used as the framework to evaluate the Malaysian healthcare financing system in terms of the provision of universal coverage for the population, and the Malaysian National Health Accounts (2008) provided the latest Malaysian data on health spending. Measuring against the four target indicators outlined, Malaysia fared credibly with total health expenditure close to 5% of its GDP (4.75%), out-of-pocket payment below 40% of total health expenditure (30.7%), comprehensive social safety nets for vulnerable populations, and a tax-based financing system that fundamentally poses as a national risk-pooled scheme for the population.

Nonetheless, within a holistic systems framework, the financing component interacts synergistically with other health system spheres. In Malaysia, outmigration of public health workers particularly specialist doctors remains an issue and financing strategies critically needs to incorporate a comprehensive workforce compensation strategy to improve the health workforce skill mix. Health expenditure information is systematically collated, but feedback from the private sector remains a challenge. Service delivery-wise, there is a need to enhance financing capacity to expand preventive care, in better managing escalating healthcare costs associated with the increasing trend of non-communicable diseases. In tandem, health financing policies need to infuse the element of cost-effectiveness to better manage the purchasing of new medical supplies and equipment. Ultimately, good governance and leadership are needed to ensure adequate public spending on health and maintain the focus on the attainment of universal coverage, as well as making healthcare financing more accountable to the public, particularly in regards to inefficiencies and better utilisation of public funds and resources.

## Introduction and background

Universal coverage and access to quality health care requires human capital, infrastructure and material resources [1]. Resources, however, are finite. With advances in medical technology, an ageing population, disease transitions, and a more demanding population, healthcare costs in middle income countries like Malaysia generally outpace the national inflation rate [2]. With the current economic downturn, the stress on national health care budgets is significant and this places people at risk of greater impoverishment due to health. To maintain an agenda for universal coverage and an equitable health system, therefore, the challenge is to develop effective structuring and management of health financing.

Malaysia has a mixed healthcare financing system. Within the private sector, private health insurance is voluntary, with variable premiums charged based on the individual’s health status, the type of health insurance, and the level of coverage. Private sector employers may elect to offer welfare and health benefits and typically negotiates packages with Managed Care Organizations (MCOs) and private insurance companies to provide medical insurance cover for their employees. Public health care services are funded through general taxation, with annual health budgets allocated by Ministry of Finance to the Ministry of Health. The proportion of general revenue allocated for Ministry of Health functions in the National Budget is decided annually [3]. In addition, the formally employed workforce make monthly contributions to an Employee Provident Fund (EPF), a compulsory savings scheme that provides a measure of security in retirement, and disburses supplementary benefits to members for medical expenses but also for capital purchases such as the family home. All private sector formal workers earning less than RM3,000 a month make a minimum contribution to the Social Security Organization (SOCSO), a scheme that provides medical benefits for work related injuries of members. Payments through SOCSO and EPF, however, do not constitute a significant proportion of healthcare expenditure because the contribution and the coverage provided are minimal. Public sector employees and their families enjoy free access to medical services provided by the public sector, and some of them have private insurance or private medical care benefits.

Most significantly, out-of-pocket expenses incurred at the point of utilization by patients, at both public and private health facilities increasingly constitute a substantial proportion of health care financing. Given the role as the custodian of health, with the responsibility to pursue universal coverage of affordable health services through an equitable and efficient health system, the Ministry of Health faces mounting pressures both internally and externally as it strives to fulfil its mandates. Similar to many countries, the Malaysian Ministry of Health has a tripartite role as a funder, provider, and regulator. Public healthcare is heavily subsidised with very low user fee (unrevised since 1982) with revenue collection is estimated to be around 2% against its spending [4]. Provision-wise, an extensive range of care is provided in the public setting with a fairly high level of geographical coverage. In terms of regulation, there is a multitude of laws, directives, and clinical practice guidelines overseeing the medical profession that also regulate medical insurance, treatment fees schedule, and the operations of private healthcare practice.

Evidences generated through global experience with different systems of health financing suggests that a strong public role in health financing, whether through payroll or general taxes, is essential for health systems to protect the poor and demonstrates that health systems with the strongest state role are likely to be more equitable and achieve better aggregate health outcomes [5,6]. Health equity, in this sense, relates to the value of fairness and just in health distribution and incorporates elements of ethics and human rights [7-11]. For health systems to function equitably towards universal coverage, financing allocations should reflect the three dimensions of coverage; the depth, breadth, and proportion of health cost covered [12]. Using Malaysia as a case study, this paper seeks to evaluate the progress and capacity of a middle income country in terms of health financing for universal coverage, and also to highlight some of the key underlying health systems challenges.

## Methods

The WHO Health Financing Strategy for the Asia Pacific Region (2010-2015) was used as the framework to evaluate the Malaysian healthcare financing system in terms of the provision of universal coverage for the population. In the absence of a set of comprehensive and internationally recognized indicators for evaluating healthcare financing for universal coverage, the WHO strategy presents the most robust basis for evaluation as it was developed in consultation with national governments and on the basis of regional health financing reviews. It is also strongly informed by growing body of global research and evidence. This regional strategy includes target indicators and strategic areas to help focus attention on key health financing issues. In addition to target indicators and strategic areas, the strategy provides more detailed and specific information on the health financing situation in the Asia Pacific region.

Specifically, the four target indicators proposed by WHO to monitor and evaluate overall progress in attaining universal coverage in countries and in the Asia Pacific region are:

1. Total health expenditure should be at least 4%-5% of the gross domestic product;

2. Out-of–pocket spending should not exceed 30-40 % of total health expenditure;

3. Over 90% of the population is covered by prepayment and risk pooling schemes; and

4. Close to 100 % coverage of vulnerable populations with social assistance and safety-net programmes.

While the indicators are developed for an overall regional perspective, they are nonetheless useful in providing baseline criteria for low and middle income countries in general, to measure their progress in terms of financing for universal coverage. Global data suggested total health expenditure within the range of 5% of GDP to be the minimum level for governments to provide adequate public infrastructure and health service delivery that could reduce catastrophic and impoverishing health expenditures [13]. Out-of-pocket payments create substantial financial barriers in accessing health care, and low-income households frequently face catastrophic health costs when out-of-pocket payments are more than 30% of total health expenditures [14]. Public financing, mainly through taxation or social health insurance or a combination of the two, is the dominant form of prepayment financing in countries that have achieved near universal coverage. Tax-based and social health insurance financing have comparative advantages and disadvantages, but both provide the risk pooling and cross-subsidization which are essential for universal coverage, access and financial protection [15] Social safety-net mechanisms aim to increase social protection by reducing barriers (economic, political, social and cultural) that can exclude the poor and vulnerable from accessing health services. Health financing oriented towards the poor can eliminate financial barriers to care by reducing out-of-pocket payments and promoting pooling that provides subsidized access for the poor.

The core source of health financing data was drawn from the most recent Malaysian National Health Accounts Report (2008) to measure up against the WHO indicators. The report follows the framework used by the Organization of Economic Cooperation and Development countries and conforms to the International Classification of Health Accounts. The System of Health Accounts (OECD, 2000 Version 1.0) has been adopted by the World Health Organization as a basis for international data collection and comparison. It proposes an integrated system of comprehensive and internationally comparable accounts and provides a uniform framework of basic accounting rules and a set of standard tables for reporting health expenditure data. The Malaysian National Health Accounts framework was based on the System of Health Accounts (OECD, 2000 Version 1.0) classification with some modifications to suit local needs. In this context, sources of financing included the public sector consisting of the federal government, state government, local authorities, and social security funds, and the private sector consisting of private health insurance, managed care organizations, household out-of-pocket expenditure, non-profit institutions, and corporations.

## Findings

The WHO recommends that for universal coverage to be achieved in Asia Pacific countries, one of the key criteria is to have adequate spending on health; a minimum total health expenditure of 4%-5% of the gross domestic product is set as the benchmark. Malaysia’s health expenditure was at 4.75% in 2008, and has been on an upward trend since 1997 (2.9%). It is important to note however that private expenditure overtook public expenditure in 2004, and in 2008 private health expenditure accounted for 53.8% compared to government health spending of 46.2%. Higher government spending is widely promoted and regarded as a means to lessen the consumption of private healthcare services that could lead to high out-of-pocket payment (OOP), as it generally relates to the provision of adequate public infrastructure and health service delivery at subsidized cost. Nevertheless, Malaysia’s relatively higher spending on health per capita GDP at USD 379 (in 2008) is decent within the developing country context, and has catered for the provision of comprehensive care with broad access and social safety nets. Among the rest of the middle income countries in the Southeast Asia region, Vietnam recorded the highest total health expenditure as percentage of GDP of 7.2% in 2009, while expenditure for Thailand, the Philippines, and Indonesia were 4.3%, 3.8%, and 2.4% respectively [16].

Another concern to universal access to healthcare services is the reliance of direct payments such user fees to providers. This is normally done through OOP which may lead to impoverishment when the payment is catastrophic. The WHO strategy recommends OOP spending to be less than 40% of the total health expenditure for its Asia Pacific region, and for Malaysia, OOP accounted for 30.7 % of the total healthcare expenditure in 2008. Except for the year 2005 and 2006, OOP has been recorded at less than 40 % of the total healthcare expenditure since 1997. Elsewhere in Southeast Asia, the rate of OOP varies significantly, with some countries in 2009 recording over 50% (The Philippines 54%, Vietnam 55%) and some less than 40% (Thailand 16%, Indonesia 35%) [16].

A closer examination of the Malaysian OOP shows that the nearly 55.0 % of the expenditure is for ambulatory care as shown in the Table 1. The expenditure incurred was for services ranging from general practitioners, specialist care, and purchase of pharmaceutical and other medical related products. Expenditures on these ambulatory care services are generally not catastrophic and they may also be reimbursed by employers (as part of staff health benefits) and private insurance. The category of OOP that raises the most concern is expenses for secondary care and hospitalization services, but this accounted for only 31.4% of the total OOP spent or 9.6% of the total health expenditure in 2008. OOPs spent in private hospitals are typically for both specialist inpatient and outpatient care, and in general are patronized by the richer class who can afford it or those who can claim reimbursements from their employers and private insurance. Nonetheless, frequent appeals in the mass media requesting for public donation for private healthcare treatment is also indicative of the existing inadequacies of public hospitals such as the lack of treatment facilities and doctors, overcrowding, and long waiting lists.

**Table 1:** Cross-classification of household out-of-pocket expenditure by provider of health services in 2008

Providers	Amount	%
Hospitals	3,388.2	31.4

Offices of Physicians	1,914.8	17.7

Offices of Dentists	202.1	1.9

Other health professions	431.4	4.0

Offices of other providers of healthcare	3,385.0	31.3

Dispensing chemist	618.2	5.7

All other sales of medical goods	746.3	6.9

All other industries as secondary producers of healthcare	117.0	1.1

**Total**	**10,803.0**	**100.0**

*Source: Malaysia National Health Accounts: Health Expenditure Report* (*2007-2008*)

The third indicator used by the WHO to monitor and evaluate universal coverage is whether over 90% of the population is covered by prepayment and risk pooling schemes. Regionally, health financing mechanisms are largely a public-private mix system at varying degrees, with countries such as Thailand providing universal health coverage through a tax-financed national health insurance system, and countries with predominant private healthcare services paid through OOP voluntary health insurance fund like the Philippines [17].

In Malaysia, citizens and residents are able to access the subsidised healthcare services provided by the Ministry of Health, university hospitals, Ministry of Defence hospitals and local authorities. The public sector healthcare services (MOH) can be considered as a national health service with its tax-based financing and heavy subsidies. In 2010, there were about 2.3 million admissions in public hospitals which accounted for about 73.2 % of the total number of admissions. Public health facilities registered about 19.2 million outpatient attendances or 87 % of the total attendances, and only a nominal sum of RM1 (approximately USD 0.30) is charged which is inclusive of medication [18]. The maximum amount that can be billed to a patient in a third class ward is RM500 (USD156) inclusive of all procedures, medication, diagnostic services and ward charges. Exemptions are also provided to those who cannot afford to pay the fees. The public facilities are also accessible to foreign workers and for the past year, unpaid hospital bills owed by foreign workers to the MOH amount to about RM18million [19]. In addition, all patients including foreign patients are exempted from any charges for infectious diseases.

The fourth recommended indicator relates to whether vulnerable populations are provided with social assistance and safety-net-programmes. Malaysia’s public healthcare system provides access to all Malaysians at a highly subsidized rate as well as geographical access to a health facility within an average 5 kilometre radius [20]. The system also caters for non-citizens including foreign workers and their families (documented or undocumented), though they have to produce a deposit or guarantor letter before hospitalization. Nevertheless, recent policy developments are encouraging all registered foreign workers (numbering up to 2 million) to subscribe to the Foreign Worker Hospitalization and Surgical Insurance Scheme, which is also a condition for the renewal of work permits. The insurance provides medical coverage up to RM10,000 yearly for an annual premium of RM120 paid by the workers themselves or employers and up to end of 2011, a total of 1.4 million foreign workers have been covered. Under the new scheme foreign workers will only need to produce their insurance card at the hospital registration counter to access public health services omitting the need for an upfront cash deposit [21].

For those in need of emergency funds for acute complex and expensive treatments, the government has set up various funds in a number of agencies such as the National Health Welfare Fund to assist these patients including those suffering from chronic diseases [20]. Non-governmental organizations and even political parties are also providing support to access these services either by providing some of these services (e.g. dialysis services by the National Kidney Foundation), subsidizing part of the payment (e.g. 1 Malaysia Fund by the Malaysian Chinese Association), or assist in appealing for public donations. In expanding the coverage of public outpatient services to underserved low income groups in densely populated areas, over 80 1Malaysia clinics were set up nationwide and operated by medical assistants and nurses offering basic outpatient care [22]. The rate is fixed similar to outpatient services in other public health facilities (RM1) for both consultation and medication for Malaysians, while non-Malaysians will be charged RM15 [23]. Up until November 2011, these clinics have recorded close to 3 million attendances [22].

## Discussion

Measuring up against WHO Asia Pacific’s 4 key proposed indicators for financing universal coverage, Malaysia has performed credibly. This can be reflected through notable health outcomes in mortality and morbidity which are on par with developed countries [17]. National vital statistics in 2009 revealed maternal mortality rate to be at 28.0 per 100,000 live births, infant mortality at 7.0 per 1,000 live births, and expected life expectancy to be 71.7 and 76.5 years for men and women respectively [18]. These outcomes are to be achieved through a comprehensive primary healthcare infrastructure that consists of an extensive rural health service, a referral system that integrates primary care clinics as the gatekeeper to secondary and tertiary care services linking district, state, regional, and general hospitals, as well as heavy government subsidy on public healthcare service provision.

Nonetheless, in moving towards attaining and maintaining a comprehensive universal coverage, various aspects beyond the health financing component needs to be taken into account. Health financing is only but one component of the overall health system strengthening framework that functions inter-connectedly with other key parts to be truly effective in delivering universal coverage and improving health outcomes. Under the WHO framework for action for strengthening health systems, five other building blocks were identified including health workforce, health information, medical products and technologies, leadership and governance, and service delivery; and all these blocks link and interact dynamically with health financing [24].

In relation to health workforce, compensation of health workers comprises a large part of health expenditures, and various provider payment methods incorporated in financing mechanisms need to be effectively used to increase health workforce motivation. The public health sector in Malaysia has always been plagued with issues of low wages and overwhelming patient loads, which has spurred the brain drain of doctors and health workers to the private sector and also outmigration [25,26]. Numerous schemes have been introduced to incentivize public health workers particularly doctors such as allowing locums, establishment of private wings, and direct salary increments but the difference with private sector remuneration benefits remains significant. In addition, unlike the public sector, there is no referral system in the private sector to screen out patients, leading to the underutilization of specialist expertise [27]. The quality of housemanship training can also be affected by the medical brain drain due to the fact that all teaching hospitals in Malaysia are run by the public sector. Nonetheless, in the last few years, the government has made inroads in developing compensation strategies which could influence and improve the health workforce skill mix to deliver priority health services, deployment, retention and performance in underserved areas.

In terms of health information, data on health expenditure that is reliably disaggregated alongside epidemiological and health outcomes data is critical to support policies and technical reviews. The collation of health information in Malaysia has been vigorous and consistent both in terms of expenditure and vital health statistics. The establishment of the National Health Accounts unit has led to the systematic documentation and production of quality health expenditure data in accordance to international standards, and these data has been instrumental in supporting evidence-based policy making. While there are known shortcomings in the lack of data from the private healthcare sector, continuous efforts have also been made to approach private sector providers to provide key information such as admission rates and expenditure levels [2]. A focused policy intervention on data sharing coupled with the development of a robust national database management system would be a viable strategy in the medium and long term to collate key missing data from the expanding private healthcare sector that would enable effective policy planning and optimization of healthcare resources.

In service delivery, health financing policies need to secure an agreed benefit package to address national health needs, especially those of the poor and including all types of care from preventive and promotive to curative and rehabilitative. While Malaysia has fared credibly in terms of curative care, there is a critical need to allocate more financing capacity to expand preventive care, in better managing escalating healthcare costs associated with the shifting of disease burden towards chronic non-communicable diseases (NCD). With NCDs dominating the top ten principal causes of death at government hospitals in 2010 [18] and also the alarming increase in the prevalence of NCDs (i.e. diabetes, obesity, stroke) [28], the effective management of chronic diseases at the population level could significantly affect the sustainability of the existing tax-based financing system. Allocative, technical and distributional efficiencies in health financing could contribute significantly to systems gains from better structure, organization and management, as well as cost-effectiveness of service delivery.

Data from the MNHA 2008 revealed that medicines and medical products constitute a significant share of out-of-pocket payments that contributed to driving the overall increase in total health expenditure. The acquisition of high-end medical equipment and new drugs should take into consideration the overall impact to the national healthcare expenditure, and purchasing decisions should be based on sound economic evaluation on population usage and cost effectiveness rather than market competition. The glut of medical equipment available in the private healthcare setting has been one of the factors that led to the increase in private health expenditure [29] where competition promotes the duplication of expensive services and equipment [26]. Issues of irrational drug use can be addressed through sound health financing policies in relation to drug procurement and distribution, alongside public education and clinical practice management. Health financing policies need to infuse the element of cost-effectiveness to better manage the purchasing of new medical supplies and equipment both in the private and public sector.

Lastly, health financing policy towards universal coverage is not isolated from the politics, pressure groups and lobbies at country level [30]. Improving the health financing system would require the critical role of the government in interacting with stakeholders and guiding the overall public interest. Good governance and leadership are needed to ensure adequate public spending on health and maintain the focus on the attainment of universal coverage, which will need to be supported by legislative and regulatory frameworks that promote prepayment and risk pooling arrangements, and rationalize health spending both in the public and private sectors. Every year, the Malaysian Auditor-General report consistently highlighted discrepancies in the management of funds and resource management of the MOH both at the federal and state level [31]. In this aspect, leadership is needed to make health care financing more accountable to the public, particularly in regards to inefficiencies and better utilisation of public funds and resources.

## Conclusion

The Member States of WHO have endorsed universal coverage as an important goal for the development of health financing systems but in order to achieve this long-term solution, flexible short-term responses are also needed. Measuring against WHO’s four target indicators for financing universal coverage, the case of Malaysia exemplifies the ways in which within a two-tier health system, notable achievements in terms of health outcomes and coverage can be attained through a mix financing system, with the government continuing to be the major financier and for those who can afford it to patronise private health services. One of the salient features of the Malaysian health system that middle income countries as well as those in the region can infer from is the critical importance of having a strong public role in health financing in protecting the poor and reaching for universal coverage.

Nonetheless, common challenges in developing countries in terms of escalating healthcare costs and expanding private healthcare continue to pose imminent risk to health equity and universal coverage. While the tax-funded public system is functioning credibly within a well-developed policy and operational framework (e.g. the civil service system), a critical systemic gap lies in the second tier, i.e. the private healthcare sector. The private realm operates in a more liberal setting and is funded by OOP, private medical insurance, and individual savings (EPF); apart from the standard laws and regulations governing medical practice and medical products, the private sector functions primarily through market mechanisms and competition. Unlike the public sector, there is no referral system in the private sector, registration of private sector specialists is voluntary, and apart from information on communicable diseases and statutory compliance for outbreaks (e.g. reporting of infectious admissions) there is no formal obligation to share health data such as daily admission rates, expenditure levels, and clinical performance.

This presents a systemic disparity in the two-tier health system which may potentially weaken the case for universal coverage on whether current achievements can be maintained in the future when uncontrolled pressures (e.g. market competition, liberalized trade, monetary incentives) in the private system start to negatively influence coverage particularly in relation to the three dimensions of universal coverage i.e. breadth and depth of coverage, and the proportion of costs covered (levels of financial risk protection). Additional prepayment financing mechanisms have been considered for the past two decades in the form of a social health insurance structure to supplement the financing of escalating healthcare costs but until today, this has not taken solid form. Health system strengthening strategies is currently underway under the proposed *1Care* health reform agenda by MOH, but its roll out has been relatively slow pending prolong internal and public consultations and detailed strategies remains unclear.

## Competing interests

The authors declare that they have no competing interests.
